# Combination of Phase Matching and Phase-Reversal Approaches for Thermal Damage Assessment by Second Harmonic Lamb Waves

**DOI:** 10.3390/ma11101961

**Published:** 2018-10-12

**Authors:** Weibin Li, Shicheng Hu, Mingxi Deng

**Affiliations:** 1 School of Aerospace Engineering, Xiamen University, Xiamen 361005, China; liweibin@xmu.edu.cn (W.L.); hscxmu@163.com (S.H.); 2 College of Aerospace Engineering, Chongqing University, Chongqing 400041, China

**Keywords:** second harmonic lamb waves, phase-reversal, phase matching, thermal damage

## Abstract

It is known that measurement and extraction of the tiny amplitude of second harmonic Lamb waves are the main difficulties for practical applications of the nonlinear Lamb wave technique. In this study, phase-reversal approaches and phase matching technique are combined to build up the second-harmonic generation (SHG) of Lamb waves. A specific Lamb wave mode pair, which satisfied phase matching conditions, is selected to ensure the generation of cumulative second harmonic waves. Lamb wave signals with the same frequency but in reverse phase, propagating in the given specimen, are added together to counteract the fundamental waves, and simultaneously to enhance the signals of the second harmonic generated. The obtained results show that the phase-reversal approach can enhance the signals of second harmonic Lamb waves, and effectively counteract that of the fundamental waves. The approach is applied to assess the thermal-induced material degradation in the stainless steel plates. Distinctions of the acoustic nonlinearity parameters under different degraded levels are clearly shown in an improved repeatable and reliable manner, while those of linear wave velocity in the specimens are neglectable. The experimental investigations performed indicate that the proposed approach can be taken as a promising alternative for assessment of material degradation in its early stages.

## 1. Introduction

Initiation and progression of material degradation accumulated via in-service thermal exposure of industrial components can significantly affect their structural integrity. The accumulation of the material degradation with increasing loads could lead to catastrophic failure without timely inspection and assessment [1,2,3]. Therefore, there is a demand to develop the nondestructive methods to assess the material degradation at an early stage to prevent interruptions of services and potential disasters that might cause loss of life or collapse of facilities. Ultrasonic guided waves, such as Lamb waves, have been widely used as the effective means for interrogating large or geometrically complex structures due to the fact that they can travel a considerable distance without scanning point by point [4]. Low energy consumption and great cost-effectiveness are the features for guided wave-based damage detection method [5]. Earlier investigations about the techniques based on Lamb waves have shown numerous successes in evaluation and inspection of discontinuities and defects over extended distances or inaccessible places in many different types of materials and structures [6,7,8,9,10]. Most Lamb wave techniques are based on linear theory and normally rely on measuring the acoustic linearity parameters such as acoustic velocity and attenuation. However, these linear Lamb wave-based approaches are less sensitive to distributed micro-damage, such as the dislocations and material degradations [11,12]. 

The use of acoustic nonlinear responses in solids is taken as a promising nondestructive testing method for micro-damage detection [13,14]. Interactions of ultrasonic waves with micro-damage in solid can cause the waveform distortion of acoustic signal, and consequently generate higher harmonics [15,16]. Thus, the received signal is composed not only of the fundamental-frequency wave but also of second harmonic generated. Considering the great advantages of Lamb waves and the high sensitivity of nonlinear acoustic measurements, nonlinear Lamb waves have been employed to detect the micro-damage in metallic plate-like structures [17,18,19,20,21,22,23]. These earlier investigations show that the use of second harmonic Lamb waves can be a promising means for detection of micro-damage in solid plates. 

However, it is found that the effect of SHG can be easily overlooked due to the dispersive nature of Lamb wave propagation [24,25,26]. Thus, one of the main difficulties for practical applications of the nonlinear Lamb wave technique, especially for the assessment of material degradation, is the relatively low efficiency of SHG (i.e., tiny amplitude of second harmonic generated). The effect of SHG induced by material nonlinearity is usually too tiny to be effectively extracted in practical applications. In addition, the signals measured including the primary (fundamental) Lamb waves and the second harmonics generated can also affect the extraction of the tiny second harmonics. Therefore, approach to enhance the signals of second harmonic generated is necessary to be developed for nonlinear Lamb wave testing. 

Phase-reversal technique (also called pulse-inversion technique) has been widely used to accentuate the contribution of even harmonics, while counteract the odd ones in medical ultrasonic imaging [27,28]. Recently, some researcher applied the phase-reversal technique of ultrasonic waves to characterize the damage in metallic materials [16,29]. However, most earlier reports focus on using phase-reversion of nonlinear ultrasonic bulk waves for nondestructive testing. There are rarely reports about nonlinear Lamb wave testing by combination of phase matching and phase-reversal approaches for damage assessment. Fukuda, et al., used pulse inversion averaging method to enhance the second-harmonic components of Lamb waves in Magnesium plates without considerations of synchronism and symmetry feature for SHG of Lamb waves [30]. Generally, nonlinear Lamb wave testing is much more complicated than that of bulk waves, for the reasons of multi-modes and dispersion nature of Lamb wave propagation. Synchronism and symmetry feature of the second harmonic generation should be firstly considered for the cumulative SHG of Lamb waves [31,32,33]. The phase matching condition (synchronism condition) for selection of Lamb wave mode pair is essentially used to counteract primary waves and accentuate second harmonic waves synchronically. The symmetric feature considered is to guarantee the desired second harmonic waves remained after applying the phase-reversal technique. 

In this paper, a phase-reversal nonlinear Lamb wave approach which combines the phase matching and phase-reversal techniques, is investigated to assess the thermal damage in stainless steel plates. A specific Lamb wave mode pair which satisfies the phase matching condition, is selected to generate the cumulative second harmonic waves. The phase-reversal approach for signal processing is used to counteract the primary wave and meanwhile to enhance the signal of second harmonic Lamb wave generated. The phase-reversal nonlinear Lamb wave approach proposed is applied to assess the thermal-induced material degradation in the 201 stainless steel plates. The correlations are presented between the acoustic nonlinearity parameters and the thermal loading time/temperature in the specimens, which show a promising alternative for assessment of material degradation. 

## 2. General Considerations

### 2.1. Principle of Phase-Reversal Approach

Material nonlinearity is essentially related to microstructure and/or presence of defects in materials. Interaction of ultrasonic wave with material nonlinearity can cause the waveform distortion of ultrasonic signal, and consequently generate higher harmonics [15,16]. One typical nonlinear phenomena considered in ultrasonic tests is the generation of second harmonic of primary wave propagation. Specifically speaking, as an ultrasonic wave propagates through a specimen with micro-damage, the interaction between micro-damage and ultrasonic wave will cause the wave distortion and thus result in the effect of second harmonic generation [34]. 

**Figure 1:**
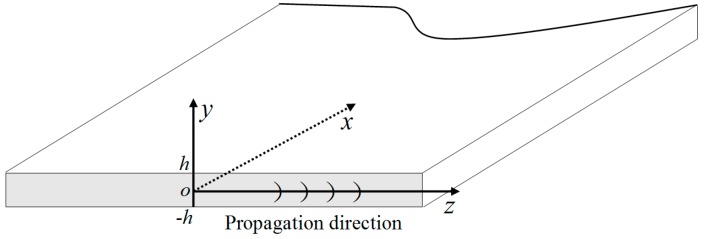
Configuration of a single elastic plate.

The acoustic field **u** of ultrasonic wave propagation can be taken as the sum of the fundamental wave $u_{\left(1\right)}$ and the second harmonic $u_{\left(2\right)}$:

(1) $$u=u_{\left(1\right)}+u_{\left(2\right)}$$

where the amplitude of $u_{\left(2\right)}$ is assumed to be much smaller than that of $u_{\left(1\right)}$. The reference configuration for analysis of Lamb wave propagation in a single elastic plate is shown in Figure 1, where the plate material is assumed to be homogeneous with no attenuation and no dispersion and with a weak elastic nonlinearity. Lamb waves propagate along the *oz* direction, and the corresponding mechanical displacements are only considered in the *yz*-plane.

Based on this consideration, one primary Lamb wave mode with a single frequency can be expressed as

(2) $$u_{\left(1\right)}=u_{\left(1\right)}(y)\exp[j(k\begin{array}{c} z \end{array}-\omega \begin{array}{c} t \end{array})],$$

where *k* and ω are, respectively, the wave number and the angle frequency, and $u_{\left(1\right)}(y)$ is the field function of primary Lamb wave. At the surface of the plate (i.e., at *y* = *h* or *y* = −*h*), the amplitude of the primary Lamb wave can be set to be *A*_1_ (i.e., formally $A_{1}=\left|\left[{u_{\left(1\right)}(y)|}_{y=h}\right]\right|$). If the Lamb wave mode pair selected (primary and double frequency Lamb waves) satisfies the conditions of phase velocity matching and nonzero power flux [11,12], the amplitude of second harmonic generated will grow with propagation distance. Accordingly, the solution to the second harmonic accompany propagation of $u_{\left(1\right)}$ can formally be given by $u_{\left(2\right)}=u_{\left(2\right)}(y)z\exp[j(2k\begin{array}{c} z \end{array}-2\omega \begin{array}{c} t \end{array})]$, where the corresponding field function $u_{\left(2\right)}(y)$ is proportional to the square of $u_{\left(1\right)}(y)$ [11,12,33,34,35]. Similarly, at the surface of the plate, the amplitude of second harmonic generated can formally be given by $A_{2}=\left|{\left[u_{\left(2\right)}(y\right)|}_{y=h}]\right|z$. The total field of Lamb wave propagation at the surface of the plate, including the fundamental wave and the second harmonic generated, is formally given by,

(3) $$u^{\left(0\right)}=A_{1}\exp[j(kz-\omega t)]+A_{2}\exp[2j(kz-\omega t)],$$

where *A*_2_ is proportional to the square of *A*_1_, and generally the magnitude of the former is much smaller than that of the latter.

If we let the phase of primary Lamb wave be reversed with that shown in Equation (2), while letting the other conditions be kept unchanged, similarly, the total field of Lamb wave propagation at the surface of the plate, including the fundamental wave and the second harmonic generated, can formally be given by,

(4) $$u^{\left(\pi \right)}=A_{1}\exp[j(k\begin{array}{c} z \end{array}-\omega \begin{array}{c} t \end{array}+\pi )]+A_{2}\exp[j(2k\begin{array}{c} z \end{array}-2\omega \begin{array}{c} t \end{array}+2\pi )],$$

By summarizing Equations (3) and (4), it is found that there is only second harmonic remained in the resultant field, i.e.,

(5) $$\tilde{u}=u^{\left(0\right)}+u^{\left(\pi \right)}=2A_{2}\exp[2j(k\begin{array}{c} z \end{array}-\omega \begin{array}{c} t \end{array})],$$

where $\tilde{u}$ is the remained field after summarizing the two Lamb wave signals in reverse phase with each other. 

**Figure 2:**
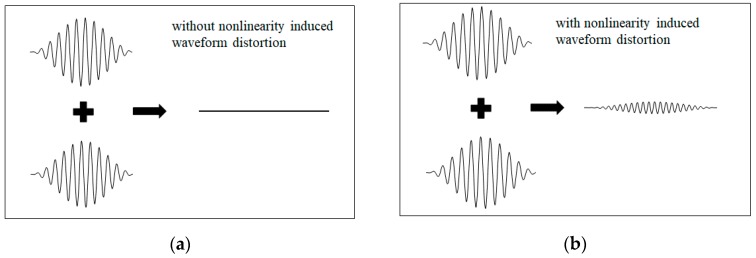
Superposition of two Lamb wave signals in reverse phase; (**a**) without and (**b**) with material nonlinearity induced waveform distortion.

The above analysis indicates that the signal of second harmonic caused by material nonlinearity for Lamb wave propagation can be enhanced by phase-reversal technique. Only the second harmonic generated remains, while the offset of the two fundamental Lamb waves in reverse phase takes place under the condition of primary Lamb wave propagation in the specimen with material nonlinearity. The mechanism of the phase-reversal approach is intuitively shown in Figure 2. Clearly, the phase-reversal approach proposed here may effectively be used to enhance the signals of second harmonic waves of primary Lamb wave propagation. 

### 2.2. Acoustic Nonlinear Parameter

The acoustic nonlinearity parameter β is introduced to quantify the effect of material degradation induced nonlinearity, which can be defined as [34,35,36],

(6) $$\beta =\frac{8}{z}\frac{A_{2}}{A_{1}^{2}}F,$$

where $A_{1}$ and $A_{2}$ are, respectively, the amplitude of the fundamental wave and the second harmonic generated, *z* is the propagation distance. *F* is a feature function, which is related to driving frequency, mode type, material property and geometric parameter of specimen. Since all these parameters are completely determined for the given measurement of Lamb waves, *F* can be regarded as a constant. In this investigation, the ratio $\beta_{R}$ = $A_{2}/A_{1}^{2}$ that is proportional to β is measured as the relative acoustic nonlinearity parameter, through which the material degradation can be quantitatively assessed [18,19,20,21]. 

### 2.3. Selection of Lamb Wave Mode Pair

In general, the effect of SHG of Lamb wave propagation is quite tiny due to its dispersion and multimodal feature [17]. Thus, it is critical to find the suitable Lamb wave mode pair to generate the cumulative second harmonic with propagation distance [17,18,19,20,21,22]. In earlier investigations, Lamb wave mode pairs satisfying the phase velocity matching and the nonzero power flux were chosen to evaluate the material nonlinearity [18,19,20,21,22,23,24]. The phase velocity matching requires that the phase velocity of the primary Lamb wave equals that of the double frequency Lamb wave, while the nonzero power flux requires that the energy transfer from the primary wave to the second-order wave generated must be nonzero [11,12,33]. From the condition of the nonzero power flux, it has been shown that in an isotropic elastic plate, the double frequency Lamb wave generated is always symmetrical regardless of whether the primary Lamb wave is symmetrical or anti-symmetrical [11,18]. Accordingly, from the dispersion curves of Lamb waves, it is convenient to find the desired Lamb wave mode pair that simultaneously satisfies both the phase velocity matching and the nonzero power flux. 

**Figure 3:**
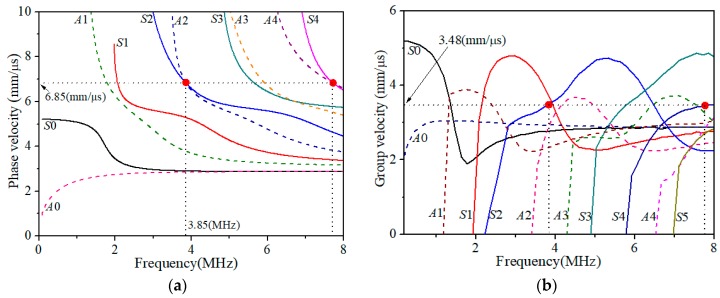
(**a**) Phase velocity and (**b**) group velocity dispersion curves for Lamb waves in the 201 stainless steel with a thickness of 1.35 mm.

For the specimens used in this investigation (1.35 mm thick stainless steel plate), the phase velocity dispersion curves of Lamb waves are shown in Figure 3a. Based on the above considerations, the mode pair ${S2|}_{f=3.85\mathrm{MHz}}$/${S4|}_{2f=7.70\mathrm{MHz}}$ or ${A2|}_{f=3.85\mathrm{MHz}}$/${S4|}_{2f=7.70\mathrm{MHz}}$ can be considered as a candidate, where the corresponding phase velocity is determined by the horizontal dashed line shown in Figure 3a, while the fundamental frequency *f* = 3.85 MHz and its double frequency 2*f* = 7.70 MHz are respectively given by the vertical dashed lines. As show in Figure 3b, the group velocities of the mode pair are also equal with each other. To guarantee the counteraction of primary waves and accentuation of second harmonic waves synchronically, the phase-reversal technique employed is only valid for the nonlinear Lamb wave testing with this kind of specific mode pair, who satisfies the both phase- and group-velocity matching conditions. 

## 3. Specimens and Experimental Setup

### 3.1. Specimens

This investigation examines the 201 stainless steel plates with chemical composition shown in Table 1. All tested specimens have the same geometrical dimension (180 mm × 30 mm × 1.35 mm) and are provided by the same supplier. The surfaces of specimens were polished and grinded to make a clean and even surface for experimental measurements. One intact specimen with the original raw material is taken as a reference. The experimental tests are conducted in two different groups of specimens subjected to thermal loading. In the first group, the thermal loading temperatures of 650 °C, 750 °C, 850 °C, 950 °C are respectively applied upon the specimens with two hours’ holding time, while, in the second group, the thermal loading temperature of 750 °C is exerted upon the specimens with a different loading time. 

### 3.2. Experimental Setup

**Figure 4:**
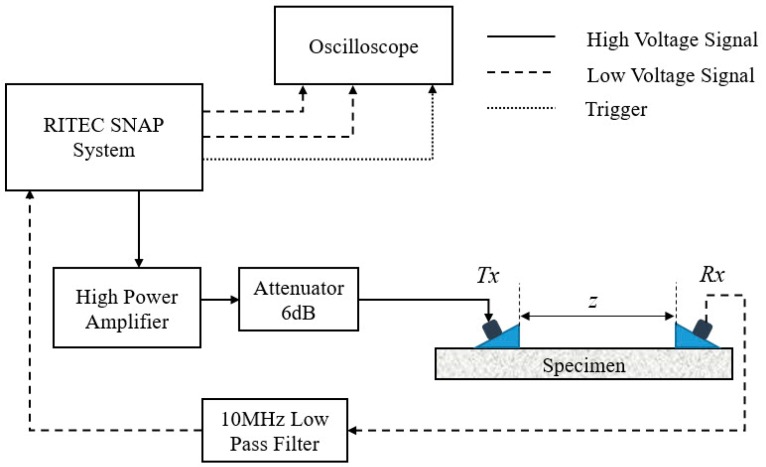
Experimental setup.

Figure 4 shows the experimental setup for measurements of the nonlinear Lamb waves. RITEC SNAP system including a high power gated amplifier is used to generate a 20-cycle sinusoidal tone-burst voltage at the frequency of 3.85 MHz. The generated sinusoidal tone-burst signal then passes through a 6 dB attenuator, which is set to purify the incident signal for a high signal-to-noise ratio (SNR). One narrow band piezoelectric transducer (as a transmitter *Tx*), whose nominal frequency is 3.5 MHz, is used to excite a longitudinal wave, and the other piezoelectric transducer (as a receiver *Rx*) with central frequency of 5.0 MHz is set for detection of primary Lamb waves and second harmonics generated. The transducers are clamped to the acrylic wedges. Light lubrication oil is used to couple the transducers to the wedges, as well as the wedges to the specimen. Although the central frequency of the receiving transducer *Rx* is 5.0 MHz, the following experimental results have shown that there are still adequate sensitivities near the driving frequency 3.85 MHz and its double frequency 7.70 MHz. 

**Figure 5:**
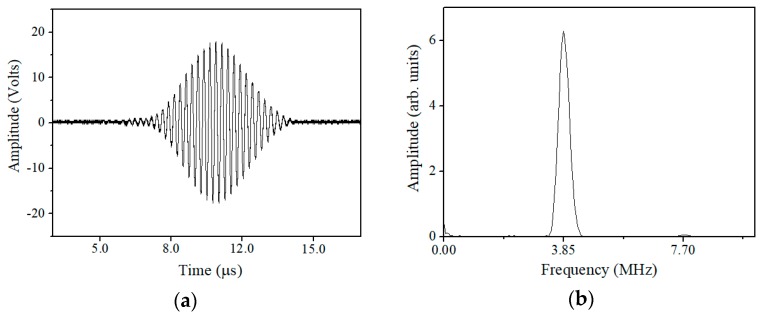
Typical excited ultrasonic signals in (**a**) time domain and (**b**) frequency spectra of the original signals.

The angle of the two wedges can be calculated by Snell’s law, once the phase velocity of Lamb waves (given by the horizontal dashed line in Figure 3a and the longitudinal wave velocity of the wedge material are given. High vacuum grease is used to acoustically couple the transducer and the wedge, as well as the wedge and the specimen. The obtained time-domain signal is processed with the fast Fourier transformation after it passes through a 10 MHz low-pass filter. Figure 5 shows a typical transmitted ultrasonic signal with a Hann window imposed on the steady-state part of the signal, and fast Fourier transform is performed on the windowed signal to get its frequency spectrum.

## 4. Measurement, Results and Discussion

**Figure 6:**
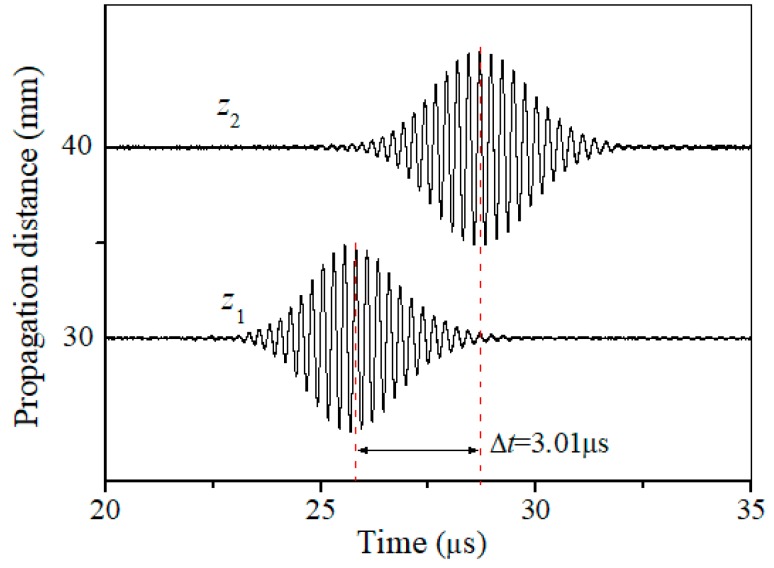
Two time-domain signals detected under different propagation distance *z*_1_ = 30 mm and *z*_2_ = 40 mm.

As shown in above analysis, it is critical to determine the phase matched Lamb wave mode pair for nonlinear Lamb wave measurements. The first setup is to verify that the signal detected is that of the Lamb wave desired. Figure 6 shows two typical time-domain signals under different propagation distance in the undamaged specimen. The group velocity of the signal detected is calculated to be $\left(z_{2}-z_{1}\right)/\Delta t$ = 3.35 mm/μs, which is very close to the theoretical group velocity (3.48 mm/μs) of the desired *S*2 mode at *f* = 3.85 MHz (RE. 5.7%) as shown in Figure 3b. It is noted that both ${S2|}_{f=3.85\mathrm{MHz}}$ and ${A2|}_{f=3.85\mathrm{MHz}}$ are excited due to the same phase velocity. However, the group velocity of the latter is obviously less than 3 mm/μs, which means the propagation speed of the wave package *A*2 is slower than that of *S*2. These two wave packages will distinguish with each other after propagating a certain distance, and the *S*2 mode will firstly been received. There is only one wave package in time domain because of the separation of two modes due to different group velocities. Thus, we are convinced that the carrier of the signals detected in Figure 6 is certainly the *S*2 mode desired. In the following experiments, investigations focus on the mode pair ${S2|}_{f=3.85\mathrm{MHz}}$/${S4|}_{2f=7.70\mathrm{MHz}}$ that may exhibit a cumulative effect for the second harmonic generated (mainly dependent on the *S*4 mode at the frequency 7.70 MHz).

**Figure 7:**
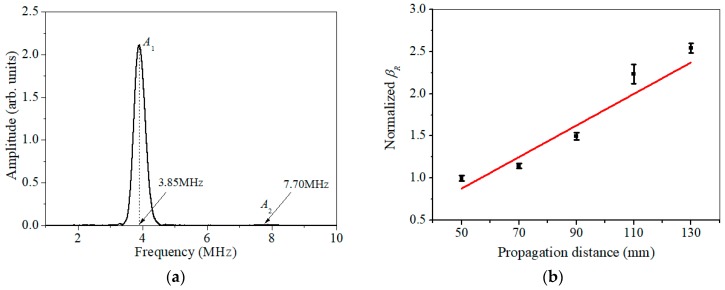
(**a**) Amplitude-frequency curve of the time-domain signal detected at *z* = 50 mm, (**b**) variation of normalized $\beta_{R}$ versus propagation distance.

It is known that the demonstration of the cumulative effect of SHG versus propagation distance is essential for nonlinear Lamb wave measurements [17,18,19,21,22]. In addition, the fact that $\beta_{R}$ grows with propagation distance can also be used to verify the measured acoustic nonlinear response is not induced from the instrumental system uncertainty, but due to the material nonlinearity. SHG can also be attributed to the nonlinearities from transducers, couplant, et al. However, only the SHG due to material nonlinearity in specimen could have the cumulative effect versus propagation distance *z* as shown in Equation (6). Figure 7a shows the amplitude-frequency curve of the time-domain signal detected at *z* = 50 mm, through which the amplitudes of the primary wave (*A*_1_) and the second harmonic generated (*A*_2_), as well as $\beta_{R}$ = $A_{2}/A_{1}^{2}$, can be completely determined. Similarly, for different spatial separation *z* in Figure 4, the corresponding $\beta_{R}$ can also be determined. Figure 7b shows that $\beta_{R}$ grows with propagation distance. Accordingly, the acoustic nonlinear response measured should be attributed to the material nonlinearity. As shown in our earlier work [34], the slope ratio of $\beta_{R}$ with wave propagation distance is used to represent the relative acoustic nonlinearity parameter to minimize nonlinearity from couplant or instrument.

**Figure 8:**
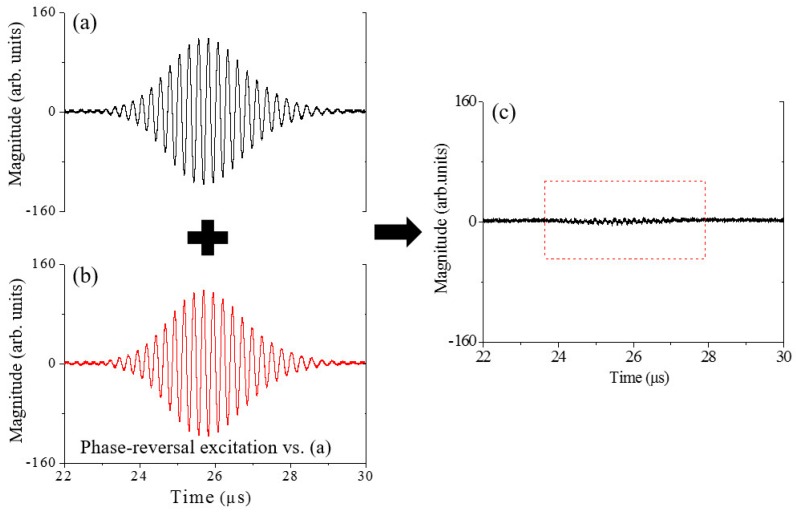
(**a**,**b**) the time-domain signals detected by *Rx* at *z* = 30 mm; (**c**) the superposition signal of the signals (**a**,**b**).

The effect of the phase-reversal approach proposed in Section 2 will be examined. Here a specific implementation process will be shown. For the undamaged specimen before thermal loading, a 20-cycle sinusoidal tone-burst voltage at the frequency of 3.85 MHz is applied to *Tx* in Figure 4, and then the time-domain signal detected by *Rx* at *z* = 30 mm is shown in Figure 8a. Next let all the conditions be kept unchanged except for a phase-reversal for the carrier of the sinusoidal tone-burst voltage applied to *Tx*, and then repeat the same measurement. For this situation, the time-domain signal detected is shown in Figure 8b. Based on the analysis given in Section 2.1, it is expected that the time-domain superposition of the two signals shown in Figure 8a,b can, to a great extent, counteract the signals out of phase (such as the fundamental waves), and enhance the signals in phase (such as the second harmonics generated). Obviously, the superposition signal shown in Figure 8c illustrates the expected effect. A software with graphical user interface is developed to compute and calculate the results for signal analysis and processing to avoid artificial errors for phase-reversal processing. The total computational time can be completed within minutes.

**Figure 9:**
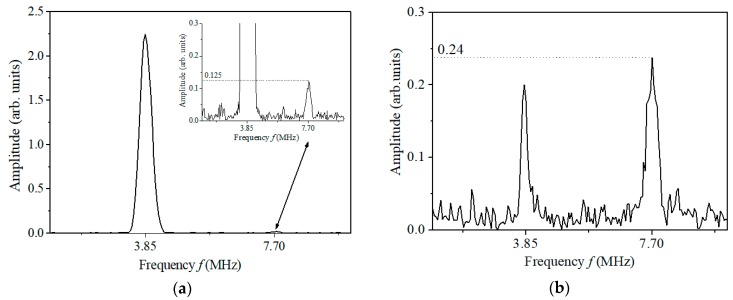
The amplitude-frequency curves measured by the conventional approach (**a**) and the phase-reversal approach (**b**).

For examining the effect of the phase-reversal nonlinear Lamb wave approach proposed, Figure 9 gives the amplitude-frequency curves of the time-domain signals shown in Figure 8a,c. As shown in Figure 9a, amplitude of the second harmonic wave is very tiny compared with that of the fundamental wave. However, by the phase-reversal approach proposed, the second-harmonic amplitude in Figure 9b is nearly twice that in Figure 9a. As indicated in Equation (5), the two fundamental waves counteract while the double-frequency component becomes twice by the phase-reversal approach. It should be noted that the amplitude of the fundamental wave does not exactly equal zero (see Figure 9b), perhaps due to the fact that the coupling conditions between the transducers and the specimen are not entirely consistent in repeated measurements. In spite of this, the phase-reversal nonlinear Lamb approach proposed can enhance the signal of second harmonic generated, and is conducive to measurements of second harmonics generated by primary Lamb wave propagation in an improved, repeatable and reliable manner. 

For a given propagation distance, the two time-domain signals similar to that shown in Figure 8a,b can be simultaneously measured using a phase-reversal excitation. For one of the two signals, the corresponding primary-wave amplitude (denoted by *A*_1*a*_) can be obtained from its amplitude-frequency curve (similar to that shown in Figure 9a. So does the primary-wave amplitude (denoted by *A*_1*b*_) of the other signal. For determination of $\beta_{R}=A_{2}/A_{1}^{2}$, *A*_1_ is calculated through $\left(A_{1a}+A_{1b}\right)/2$, which may inhibit the influences of the instrumental transient response or coupling conditions in repeated measurements (note the nonzero amplitude for the primary wave in Figure 9b). Meanwhile, *A*_2_ is acquired through the amplitude- frequency curve of the superposition signal similar to that shown in Figure 8c (see the second-harmonic amplitude in Figure 9b). Referring to the measurement process described here, the corresponding $\beta_{R}$ can be determined for the specimens with different damage levels. Specifically, the phase-reversal nonlinear Lamb wave approach proposed is applied for tracking the thermal induced degradation in the stainless steel plates. 

**Figure 10:**
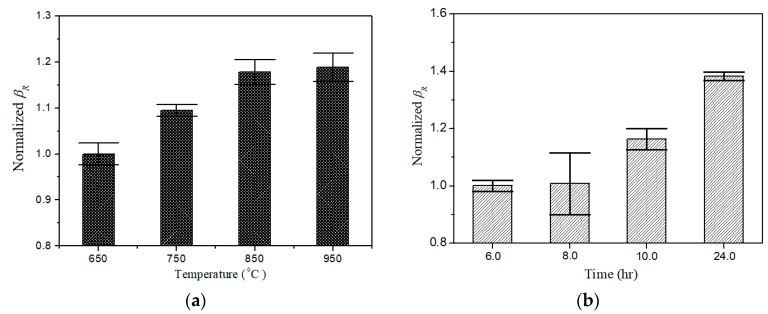
Variation of normalized for the specimens subjected to (**a**) thermal loading temperature with two hours’ holding time (by the reference of $\beta_{R}$ = 0.197, that of the specimen subjected 650 °C thermal loading), and (**b**) different thermal loading time at the temperature of 750 °C (by the reference of $\beta_{R}$ = 0.363, that of the specimen subjected 6 hours’ thermal loading).

In the present experimental investigations, different degradations of the specimens suffered from different thermal loadings are artificially controlled (see Section 3.1). Using the mode pair ${S2|}_{f=3.85\mathrm{MHz}}$**/**${S4|}_{2f=7.70\mathrm{MHz}}$ (see Figure 3) and following the phase-reversal approach described above, the value of $\beta_{R}$ can readily be determined for the specimens subjected to different thermal loadings. Figure 10a,b shows the variation of normalized $\beta_{R}$ for the specimens, respectively, subjected to different thermal loading temperatures with two hours’ holding time and different thermal loading time at the temperature of 750 °C.

**Figure 11:**
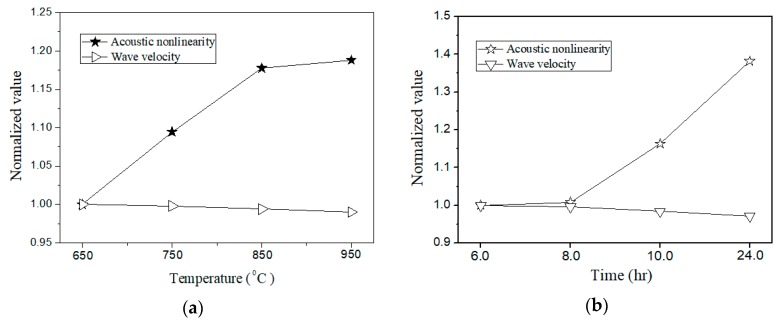
Comparison of the sensitivity of acoustic nonlinearity and linear parameters for the specimens subjected to (**a**) thermal loading temperature with two hours’ holding time (by the reference of $\beta_{R}$ = 0.197, *C_g_* = 3.346 mm/μs that of the specimen subjected 650 °C thermal loading), and (**b**) different thermal loading time at the temperature of 750 °C (by the reference of $\beta_{R}$ = 0.363, *C_g_* = 3.332 mm/μs that of the specimen subjected 6 hours’ thermal loading).

Here the variation of acoustic nonlinearity (described by $\beta_{R}$) versus different thermal damage levels is obtained to verify the validity of the approach proposed. It is found that the normalized $\beta_{R}$ increase with either the thermal loading time or the thermal loading temperature. The results shown in Figure 10 indicate that the phase-reversal nonlinear Lamb wave approach is a feasible method to enhance the signal of second harmonic generated, and thus increases the reliable capacity (due to high SNR second-harmonic signals measured) for assessment of material degradation in specimens. As shown in Figure 11, group velocities (described by *C*_g_) of Lamb wave in the specimens with and without thermal loadings are also provided to compare the sensitivity of linear and nonlinear parameter. It is clearly shown that the differences of wave velocity can be neglected, which indicates that the linear Lamb wave based evaluation technique is insufficiently to evaluate the thermal induced degradation in early stage. In this study, all measured linear and nonlinear parameters are normalized by the values of the raw material to display only the relative change. Consequently, these results indicate that nonlinear ultrasonic parameters are much more promising with significantly improved sensitivity over linear parameters for early stage detection of thermal damages in this specimen.

## 5. Conclusions

In this study, a combination of phase matching and phase-reversal approaches is investigated to measure second harmonic Lamb waves for thermal damage assessment. For the given specimen, a specific Lamb wave mode under the phase and group-matching conditions is selected to ensure the generation of cumulative second harmonic. Two Lamb wave signals, with the same frequency but in reverse phase, are added together to counteract the fundamental waves, and meanwhile to enhance the second harmonic wave. The experimental investigation performed does verify the expected effect of signal enhancement of SHG by primary Lamb wave propagation. On this basis, the proposed approach is applied to assess the thermal-induced degradation in the stainless steel plates. The correlation between the relative acoustic nonlinearity parameter and the thermal-induced degradation (induced by thermal loading temperature/time) in the test specimens are obtained. Distinctions of the relative acoustic nonlinearity parameters for the specimens under different degraded levels are clearly provided. A comparison of the linear and nonlinear ultrasonic parameters variation in different specimens is also conducted. It is found that the differences of linear parameter in the specimens are neglected. The experimental investigations performed indicate that the proposed phase-reversal nonlinear Lamb wave approach can be taken as a promising alternative for assessment of material degradation in metallic specimens. It is important to note that the counteraction of primary Lamb waves and accentuation of second harmonic Lamb waves must be synchronic. Thus, phase and group matching conditions of the primary Lamb wave and second harmonic one should be satisfied precisely to ensure the accuracy of the obtained results. In addition, stable and consistent electrical signals with opposite phase excited at different times are also important to obtain the accurate results. 

## Figures and Tables

**Table 1:** Chemical composition (wt %) of 201 stainless steel.

C	Si	Mn	Cr	N	P	Ni	Cu	Fe
≤0.15	≤0.75	5.5–7.5	16–18	≤0.25	≤0.06	3.5–5.5	2.3	Remain
